# Comparing the activity of broad-spectrum beta-lactams in combination with aminoglycosides against VIM-producing *Enterobacteriaceae*

**DOI:** 10.1128/spectrum.03876-23

**Published:** 2024-08-20

**Authors:** Justin A. Clark, David S. Burgess

**Affiliations:** 1 College of Pharmacy, University of Kentucky, Lexington, Kentucky, USA; JMI Laboratories, North Liberty, Iowa, USA

**Keywords:** Verona integron-encoded metallo-beta-lactamase, carbapenem resistance, *Enterobacteriaceae*, antimicrobial susceptibility, time-kill, beta-lactams, aminoglycosides

## Abstract

Metallo-beta-lactamase (MBL)-producing carbapenem-resistant *Enterobacteriaceae* (CRE) infections continue to pose a serious threat to healthcare. Due to their unique active site, MBLs evade the activity of many novel beta-lactam/beta-lactamase inhibitor combinations, which have been specifically targeted toward those carbapenemases with serine active sites. Furthermore, resistance to most, if not all, other clinically relevant antimicrobial classes leaves few reliable therapeutic options. Combination therapy has thus played a vital role in the treatment of MBL-producing CRE infections. In this study, we utilized the static time-kill assay to investigate clinically relevant concentrations of cefepime, piperacillin-tazobactam, and meropenem alone and in combination with either amikacin or the novel plazomicin to determine if combinations of routinely used beta-lactam therapy with an aminoglycoside would achieve bactericidal activity against eight clinically isolated Verona integron-encoded MBL (VIM)-producing CRE. Furthermore, we compared this activity to the combination of aztreonam/avibactam, which has shown potent activity against MBL-producing CRE. Both aztreonam/avibactam and meropenem with either aminoglycoside were rapidly bactericidal within 4 hours and remained bactericidal through 24 hours against all isolates with few exceptions. Combinations including cefepime and piperacillin-tazobactam were also rapidly bactericidal, but activity after 24 hours was inconsistent depending upon the partner aminoglycoside and isolate. Further investigation is warranted to elucidate optimal antibiotic exposures against MBL-producing CRE, including novel agents in the pipeline.

**IMPORTANCE:**

Carbapenem-resistant *Enterobacterales* (CRE) are one of the most pressing antimicrobial-resistant threats at present. In addition to exhibiting resistance to many, if not all, commonly used antimicrobial agents, CRE achieves these resistant phenotypes through a variety of mechanisms, each of which can uniquely affect available treatment options. The present study is an *in vitro* investigation of several Verona integron-encoded metallo-beta-lactamase (VIM)-producing CRE isolated from patients at our academic medical center. Because metallo-beta-lactamases (MBLs) are inherently resistant to many of the novel treatments designed to treat CRE due to their different active site composition, we tested several antimicrobial combinations containing routinely utilized broad-spectrum beta-lactams and aminoglycosides. Our results further our understanding of combination therapy options against VIM-producing CRE, including with non-carbapenem-beta-lactams cefepime and piperacillin. By optimizing combinations of existing antimicrobial agents, we hope to expand the available armamentarium against these resistant pathogens.

## INTRODUCTION

Antibiotic resistance remains one of the most pressing issues in healthcare. Recent estimates from the Centers for Disease Control and Prevention Antibiotic Resistance Threats Report show that each year more than 2.8 million infections with an antibiotic-resistant pathogen led to more than 35,000 deaths in the United States alone (1). Listed among the most serious (urgent) threats are carbapenem-resistant *Enterobacteriaceae* (CRE), which has been recognized as an urgent threat since the initial Antibiotic Resistance Threats Report in 2013 (2). CRE was estimated to cause 13,100 hospitalized cases in 2017 leading to an estimated 1,100 deaths and $130 million in associated healthcare costs. Despite increased surveillance efforts and implementation of preventative protocols, the estimated number of annual cases has remained stable between 2012 and 2017, which demonstrates a need for continued improvements.

Improvements to the antimicrobial armamentarium against CRE have been spearheaded by novel beta-lactam/beta-lactamase inhibitor combinations. Ceftazidime/avibactam (Avycaz), meropenem/vaborbactam (Vabomere), and imipenem/cilastatin/relebactam (Recarbrio) are most notably active against *Klebsiella pneumoniae* carbapenemase (KPC), which belong to Ambler Class A; however, they also possess activity against Class C, and ceftazidime/avibactam extends activity against some Class D carbapenemases (3). The addition of aztreonam to ceftazidime/avibactam further extends the activity of the combination to Class B carbapenemases, which differ from Classes A, C, and D based on their active site composition [metallo-beta-lactamases (MBL) vs serine-based beta-lactamases]. Plazomicin (Zemdri), a novel aminoglycoside, also claims activity against all CRE phenotypes as it belongs to a different antimicrobial class. The availability of these new agents has removed the need for riskier, older agents like colistin and polymyxin B.

The objective of this study was to compare the activity of several, routinely utilized beta-lactams (aztreonam, cefepime, meropenem, and piperacillin/tazobactam) and aminoglycosides (amikacin and plazomicin) against Verona integron-encoded MBL (VIM)-producing CRE. Firstly, we investigated each of these antimicrobials as monotherapy exposures. We also combined cefepime, meropenem, and piperacillin/tazobactam with each aminoglycoside. Lastly, we investigated the novel beta-lactamase inhibitor avibactam in combination with aztreonam.

## MATERIALS AND METHODS

### Isolates

All eight isolates were cultured from patients at our academic medical center from 2011 to 2019 during routine clinical care. Most of these isolates were *Enterobacter cloacae* (EC_53, EC_134, EC_167, EC_169, EC_416, and EC_608) and two were *K. pneumoniae* (KP_173 and KP_411). These isolates were selected principally based on meropenem minimum inhibitory concentrations (MICs ≥ 16 µg/mL). The Gram-negative blood culture (BC-GN) nucleic acid test (Nanosphere, Inc., Northbrook, IL) was utilized to detect the presence of and identify the metallo-beta-lactamases. Additionally, we utilized a Kirby Bauer disk diffusion method described by Tsakris et al. to confirm metallo-beta-lactamase expression (4).

### Susceptibilities

Broth microdilution (BMD) was utilized to obtain MICs per CLSI recommendations (5). Antimicrobials (amikacin, aztreonam, aztreonam/avibactam, cefepime, meropenem, piperacillin/tazobactam, and plazomicin) were dissolved in Cation-adjusted Mueller Hinton Broth (CAMHB) and serially diluted in 96-well trays. All beta-lactamase inhibitors were plated at a flat concentration of 4 µg/mL.

### Time-kill assay

Static concentration time-kill assays were utilized to assess the activity of antimicrobial exposures against the CRE isolates. For all isolates tested, experiments were conducted with a growth control (GC) absent of antimicrobials in addition to amikacin (AMK), aztreonam (ATM), aztreonam/avibactam (ATM-AVI), cefepime (FEP), meropenem (MEM), piperacillin/tazobactam (TZP), plazomicin (PLZ), cefepime/amikacin (FEP-AMK), meropenem/amikacin (MEM-AMK), piperacillin/tazobactam/amikacin (TZP-AMK), cefepime/plazomicin (FEP-PLZ), meropenem/plazomicin (MEM-PLZ), and piperacillin/tazobactam/plazomicin (TZP-PLZ) exposures. The concentrations utilized for each of these antimicrobials are as follows: AMK 4 µg/mL, ATM 32 µg/mL, FEP 32 µg/mL, MEM 16 µg/mL, TZP 64 µg/mL, and PLZ 4 µg/mL. All beta-lactamase inhibitors were added at a flat concentration of 4 µg/mL. These concentrations were chosen to simulate the steady-state concentration of each exposure within a continuous infusion regimen. We utilized the following relationship to calculate our concentrations:


$$C_{ss}=\frac{X_{d}}{Cl_{tot}},$$

where C_ss_ is the steady state concentration, X_d_ is the total daily dose, and Cl_tot_ is the total systemic clearance. The following continuous infusion regimens were simulated: aztreonam (± avibactam) 2,000 mg every 8 hours, cefepime 2,000 mg every 8 hours, meropenem 2,000 mg every 8 hours, and piperacillin/tazobactam 4,500 mg every 6 hours. For the aminoglycosides, conservative estimates of the expected exposures of amikacin 15 mg/kg and plazomicin 15 mg/kg (assuming 70 kg) every 24 hours were utilized. Population systemic clearance terms were obtained from previously published clinical trials (6–11).

As with the BMD procedure, CLSI recommendations were followed in conducting the time-kill procedure (12). Briefly, flasks containing CAMHB were inoculated with 0.5 McFarland matched bacterial suspensions to target an initial bacterial concentration of 7.5 × 10^5^ CFU/mL and stored at 35°C with agitation. Serial samples were taken at 0 (immediately after inoculation), 4, 8, and 24 hours, and logarithmically spiral plated on Mueller Hinton Agar plates. Inoculated plates were stored at 37°C between 16 and 24 hours after which the colonies were counted. Counts were censored below 10^2^ (lower limit of detection) and above 10^10^ (maximum count) by rounding either up/down, respectively. All experiments were performed in duplicate. If the results differed categorically (bactericidal, bacteriostatic, or regrowth), the experiment was performed a third time.

### Statistical analysis

The primary outcome assessed was the mean total reduction in bacterial concentration (CFU/mL) between each antimicrobial exposure and GC after 24 hours. Other outcomes include a subgroup analysis of mean total bacterial reduction in plazomicin vs amikacin and plazomicin vs aztreonam/avibactam exposures. Also, categorical comparisons of synergy, indifference, and antagonism and bactericidal, bacteriostatic, and regrowth at 24 hours were assessed. Comparisons of mean bacterial reduction were conducted with repeated measures analysis of variance using Tukey’s method for pairwise comparison adjustment to control the family-wise error rate. Alpha was defined a priori as 0.05. All statistics and figures were generated using Python (v3.5).

## RESULTS

### Isolates

VIM was the only carbapenemase identified by Nanosphere in all the included isolates except for EC_134, in which KPC was additionally identified. The susceptibilities for all isolates are included in Table 1. All isolates were resistant to the four beta-lactam agents tested, particularly to the non-carbapenem agents. Conversely, all isolates were susceptible to aztreonam/avibactam and both aminoglycosides. Of note, the MICs measured for plazomicin and aztreonam/avibactam were susceptible by a wide margin, while the isolates susceptible to amikacin were either equal to or a single log_2_ dilution below the CLSI susceptibility breakpoint (5).

**TABLE 1:** Susceptibilities for clinical isolates included in study^*a*^

Isolate	AMK	ATM	ATM/AVI	FEP	MEM	TZP	PLZ
EC_53	16	256	0.13	128	16	512	0.06
EC_134	8	256	0.06	128	32	512	0.25
EC_167	8	128	0.06	64	16	512	0.25
EC_169	8	256	0.06	128	16	512	0.25
EC_416	16	16	0.06	64	32	>512	0.25
EC_608	16	>256	0.125	128	16	>512	0.5
KP_173	16	16	≤0.03	32	16	>512	0.5
KP_411	8	32	0.06	256	32	>512	0.5

^*a*^ MICs are reported in units of μg/mL. Isolates with the EC prefix are *E. cloacae* and KP are *K. pneumoniae*.

### Starting inoculum and controls

The average bacterial concentrations for all antimicrobial agents can be found in Table 2. Overall, the average bacterial concentration measured across all exposures at baseline was 5.85 ± 0.12 log_10_ CFU/mL. Furthermore, the growth controls reached the maximum concentration measured after 4 hours and sustained for the duration of the experiments (9.84 ± 0.32, 10 ± 0.0, 10 ± 0.0, respectively).

**TABLE 2:** Bacterial concentration for all sampled time points^*a*^

Drug conditions	T0	T4	T8	T24
AMK	5.89 ± 0.1	4.93 ± 1.62	6.32 ± 1.97	9.88 ± 0.28
ATM-AVI	5.86 ± 0.09	2.95 ± 0.6	2.22 ± 0.26	2.0 ± 0.0
ATM	5.86 ± 0.07	6.64 ± 2.95	7.99 ± 2.64	8.9 ± 2.79
FEP	5.9 ± 0.1	4.1 ± 0.89	6.85 ± 2.72	9.64 ± 0.94
FEP-AMK	5.87 ± 0.09	3.69 ± 0.26	2.76 ± 0.65	5.04 ± 3.42
FEP-PLZ	5.86 ± 0.12	3.21 ± 1.66	3.09 ± 1.53	2.7 ± 1.09
GC	5.86 ± 0.1	9.84 ± 0.32	10.0 ± 0.0	10.0 ± 0.0
MEM	5.89 ± 0.11	2.45 ± 0.38	2.47 ± 0.79	3.6 ± 2.65
MEM-AMK	5.89 ± 0.1	2.86 ± 0.38	2.05 ± 0.14	2.0 ± 0.0
MEM-PLZ	5.87 ± 0.13	2.76 ± 1.13	2.55 ± 0.99	2.23 ± 0.52
PLZ	5.87 ± 0.12	3.2 ± 1.68	3.1 ± 1.55	2.76 ± 1.14
TZP	5.88 ± 0.11	6.99 ± 1.99	9.97 ± 0.07	9.87 ± 0.19
TZP-AMK	5.88 ± 0.07	4.08 ± 0.34	3.98 ± 1.5	7.41 ± 2.97
TZP-PLZ	5.88 ± 0.15	3.26 ± 1.63	3.11 ± 1.51	2.68 ± 1.06

^*a*^ Bacterial concentrations (log_10_ CFU/mL) are reported as geometric mean ± standard deviation.

### Comparison to GC

**Fig 1:**
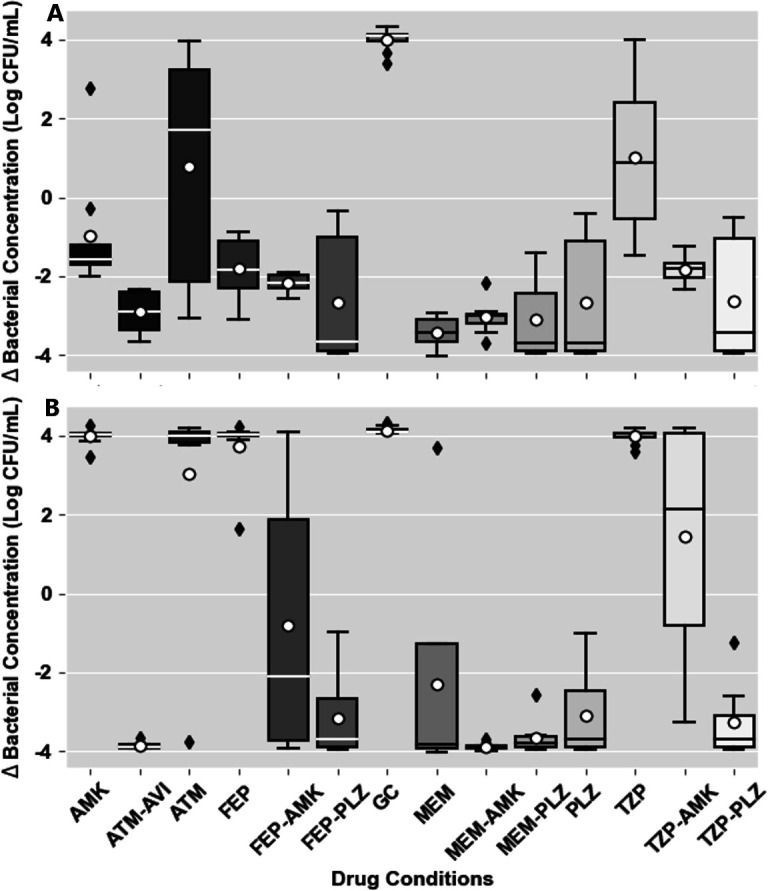
Above are the boxplots illustrating the distributions of log bacterial reduction following (A) 4 hours and (B) 24 hours of exposure. Outliers are denoted as diamonds above/below the upper/lower fence, and geometric means are denoted as white circles.

The average bacterial concentration changes from baseline can be found in Table S1. The distributions of these changes are illustrated in more detail after both 4 and 24 hours in Fig. 1 and after 8 hours in Fig. S1. When considering the relative differences of these concentration changes between the growth control and each agent tested, most of the monotherapy exposures did not significantly differ from growth control (regrew) after 24 hours of exposure (MEM and PLZ were the exceptions). All combination exposures were significantly different after 24 hours except TZP-AMK, which showed regrowth. These mean differences are included in Table 3.

**TABLE 3:** Comparison of bacterial concentration changes between antimicrobial exposure and growth control after 24 hours^*a*^

Drug conditions	Mean difference [95% CI]	*P* value
AMK	−0.16 [−3.11, 2.78]	0.9
ATM-AVI	−8.01 [−10.96, –5.06]	<0.001
ATM	−1.11 [−4.05, 1.84]	0.9
FEP-AMK	−4.97 [−7.91, –2.02]	<0.001
FEP-PLZ	−7.3 [−10.25, –4.36]	<0.001
FEP	−0.4 [−3.34, 2.55]	0.9
MEM-AMK	−8.04 [−10.98, –5.09]	<0.001
MEM-PLZ	−7.79 [−10.73, –4.84]	<0.001
MEM	−6.44 [−9.39, –3.49]	<0.001
PLZ	−7.25 [−10.2, –4.3]	<0.001
TZP-AMK	−2.71 [−5.66, 0.24]	0.1329
TZP-PLZ	−7.42 [−10.37, –4.48]	<0.001
TZP	−0.16 [−3.11, 2.79]	0.9

^*a*^ Mean difference was calculated as drug conditions—GC. Groups compared aggregated bacterial concentration (log_10_ CFU/mL) differences across each respective experiment.

### Cidality

**Fig 2:**
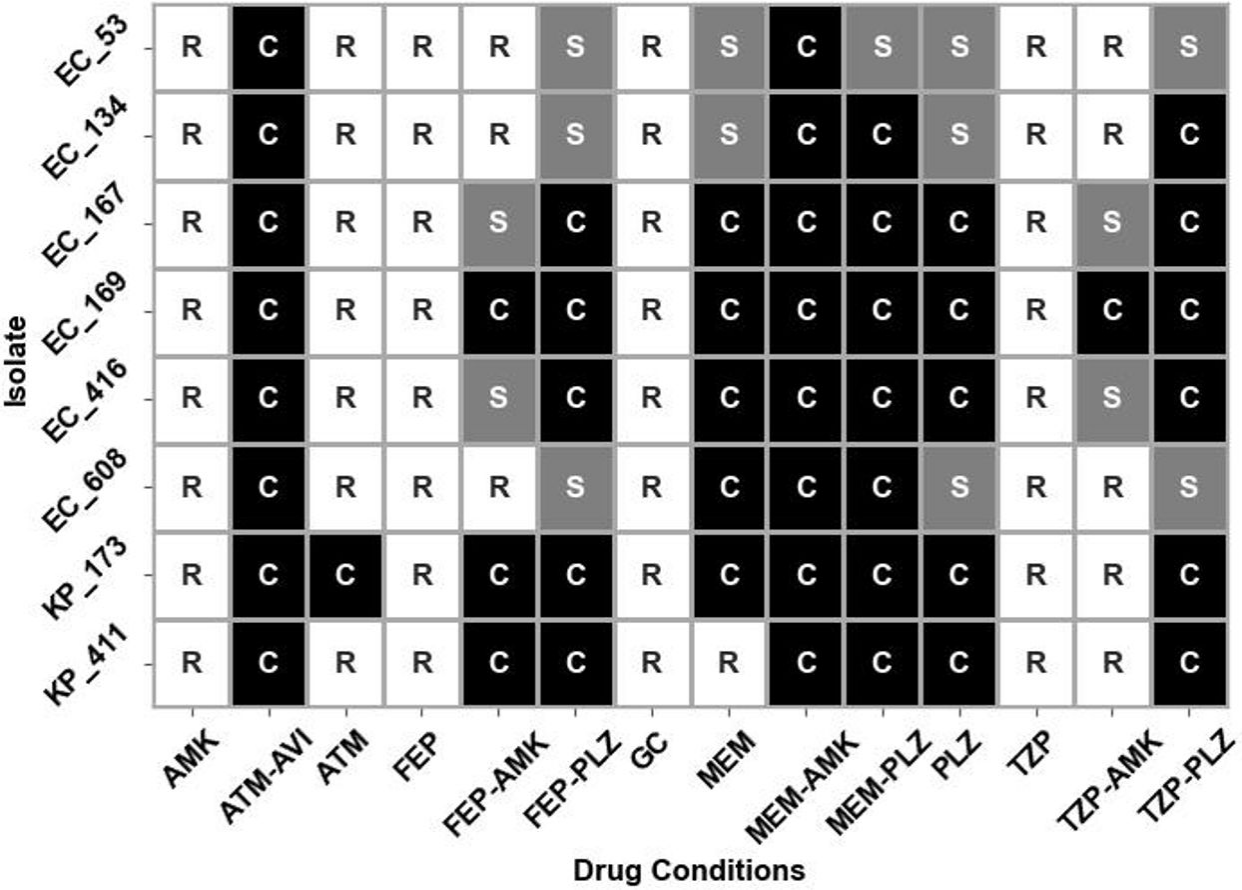
Illustrated is a heatmap showing the categorical bactericidal analysis for each drug combination against all isolates tested. The average bacterial concentration differences measured across all duplicate trials for each isolate were utilized for this assessment. C: bactericidal, S: bacteriostatic, R: regrowth.

All the combination exposures that significantly differed from growth control were at least bacteriostatic on average after 24 hours, and all but one (FEP-AMK) were bactericidal (Fig. 1). These relationships are illustrated for each isolate in Fig. 2. In addition to the combination exposures, PLZ monotherapy was also bactericidal after 24 hours, and MEM monotherapy was bacteriostatic after 24 hours. When considering the median bacterial concentration reduction, MEM would be considered bactericidal.

### ATM/AVI vs PLZ vs AMK

The differences in activity between the aminoglycosides are notable. On average, PLZ alone was bactericidal after 24 hours, while AMK regrew to the maximum measured value leading to a large difference between the two [mean difference (95% CI): 7.09 (4.14, 10.03)]. MEM-AMK was the most active exposure tested and was the only bactericidal amikacin-containing exposure (mean difference from T0 ± std: −3.89 ± 0.1); although, FEP-AMK remained bacteriostatic after 24 hours (mean difference from T0 ± std: −0.82 ± 3.37). The activity of MEM-PLZ and MEM-AMK were strongly similar after 24 hours, with MEM-AMK having a marginally larger bacterial reduction [mean difference (95% CI): −0.25 (−3.19, 2.7)]. While FEP-PLZ and TZP-PLZ were much more active than the AMK combinations, the beta-lactams added little to the activity of PLZ monotherapy after 24 hours [mean difference (95% CI): 0.05 (−2.89, 3.00) and 0.10 (−2.84, 3.05), respectively]. ATM-AVI showed similarly strong activity to MEM-AMK (mean difference from T0 ± std: −3.86 ± 0.09). Both meropenem-containing combinations were the only exposures to produce 3 log_10_ bacterial concentration reduction at all sampled time points, although ATM-AVI and all other plazomicin-containing exposures nearly accomplished the same (−2.63 ± 1.54 was the highest point estimate across any time point for these exposures). All mean differences between amikacin- and plazomicin-containing exposures and aztreonam/avibactam can be found in Tables S2 and S3.

### Synergy

Amikacin combinations exhibited more synergy than plazomicin combinations after 24 hours. All amikacin combinations were synergistic against at least 3/8 isolates, with FEP-AMK exhibiting synergy most frequently (6/8 isolates). None of the plazomicin combinations exhibited synergy, owing to the strong activity of plazomicin monotherapy. ATM-AVI exhibited synergy against all but a single isolate (KP_173), in which the activity of aztreonam mirrored that of aztreonam/avibactam after 24 hours (mean difference from T0 ± std: −3.77 ± 0.09 and −3.85 ± 0.03, respectively). The overall synergy categorical analysis is illustrated in Fig. S2.

## DISCUSSION

We tested combinations of routinely used beta-lactams for Gram-negative empiric therapy in combination with both a novel and long-existing aminoglycoside in addition to the combination of aztreonam and avibactam. Utilizing clinically relevant doses, we noted consistent bactericidal activity for aztreonam/avibactam, all plazomicin exposures, and meropenem/amikacin against 8 VIM-producing carbapenem-resistant *E. cloacae* and *K. pneumoniae*.

Combination therapy, specifically with a beta-lactam and an aminoglycoside, has long been supported by *in vitro* data (13). We have provided additional evidence of this relationship with both amikacin and plazomicin-containing combinations. In previous studies published by our lab, Kulengowski et al. demonstrated bactericidal activity against KPC-, VIM-, and KPC/VIM-producing isolates using combinations of meropenem and amikacin at similar concentrations utilized in our present study; however, isolates with more elevated MICs to both meropenem and amikacin overcame the combination (14, 15). Other investigators have demonstrated either bacteriostatic or bactericidal activity for meropenem and amikacin against carbapenemases of all Ambler classes, though the various concentrations of meropenem and amikacin differed (16–18). In our present study, we provide additional evidence of this combination, with every experiment resulting in bactericidal activity with this combination. Meropenem/amikacin was numerically the most active exposure tested, though it did not strongly differ from several other highly active combinations.

When using cefepime or piperacillin/tazobactam in combination with amikacin, the activity was much more erratic. The cefepime and amikacin combination was bactericidal in at least two experiments for 5/8 isolates, while piperacillin/tazobactam only achieved this in 2/8 isolates. While it appears that a carbapenem combined with amikacin can provide activity against various carbapenem phenotypes, other routinely used broad-spectrum beta-lactams will be unreliable until we have more targeted information to distinguish when they will or will not be effective.

Plazomicin is a novel aminoglycoside that was rationally designed to evade the most clinically relevant aminoglycoside-modifying enzymes (19). Unlike amikacin, plazomicin has been shown to have potent activity in various CRE phenotypes in combination with both meropenem and non-carbapenem beta-lactams (piperacillin/tazobactam and ceftazidime) (20–22). While these studies dosed plazomicin in multiples of the MIC, our concentrations were much higher than most of the experiments reported. At these higher concentrations, we noted bactericidal activity for plazomicin monotherapy for 5/8 isolates. The combinations of cefepime and piperacillin/tazobactam with plazomicin resulted in comparable results to plazomicin monotherapy, suggesting that plazomicin was solely driving the activity. Furthermore, the combination of meropenem with plazomicin predictably resulted in potent bactericidal activity similar to what was observed with the combination of meropenem with amikacin.

Because aztreonam intrinsically resists hydrolysis mediated by MBLs, the combination of this agent with one of the novel inhibitors restores the activity of aztreonam in MBLs co-harboring additional beta-lactamases. Aztreonam/avibactam was among the most active combinations we tested, which achieved bactericidal reductions by 8 hours in all experiments and was maintained through 24 hours. This is starkly contrasted with the activity of aztreonam monotherapy, which was only bactericidal in KP_173. This was the result of KP_173 not possessing any other beta-lactamases with the ability to hydrolyze aztreonam. Numerous other investigators have demonstrated the effectiveness of aztreonam/avibactam against MBL-producing CRE isolates, though many of these studies have focused on the New Delhi metallo-beta-lactamase phenotype (23–25).

The potent activity of meropenem monotherapy was an unexpected result. Most of the isolates (6/8) had meropenem MICs of 16 µg/mL, which at best should have resulted in bacteriostatic activity. Of the three isolates with higher MICs (all 32 µg/mL), only KP_411 showed consistent regrowth when exposed to meropenem. Furthermore, EC_134 regrew completely at 24 hours in only 1/3 experiments. In the other two experiments, and both EC_416 experiments, meropenem showed bactericidal activity at 24 hours. EC_53 was the only other isolate to ever regrow when exposed to meropenem. Although we do not have a definitive explanation for this behavior, one possibility may be within strain variability. While the reported modal MICs are all either equal to or 1 log_2_ dilution higher than the tested concentration of meropenem, isolate subcultures selected during these experiments may have exhibited MICs below the modal value. Care should be taken when extrapolating our results, especially in VIM-producing isolates with higher meropenem MICs, as these data may not extrapolate to such scenarios.

One of the limitations of this study is the absence of data for cefiderocol. Cefiderocol has demonstrated activity against *Enterobacteriaceae* expressing Class A–D beta-lactamases. We tested MICs of 19 MBL-producing *K. pneumoniae* and *E. cloacae* (all VIM) using MIC strips, and 18/19 isolates, including all nine isolates tested in this study, were susceptible to the CLSI breakpoint ≤4 µg/mL (J. A. Clark, D. S. Burgess, unpublished data). Another limitation present in the study is the use of only the static time-kill method. This simulation of a continuous infusion provides a best-case scenario for the beta-lactam agents tested; however, the aminoglycosides are best utilized when maximizing both the peak/MIC and MIC/AUC ratios. Lastly, we tested a small number of isolates from a single medical center, so these results should be contextualized with local resistance patterns when considering their application to other centers.

Now that we have options to treat infections caused by MBL-producing CRE, it is vital we optimize these regimens to ensure the best patient outcomes. Furthermore, optimizing combinatory exposures of both existing and novel agents will lead to rational selection of partner antimicrobials to overcome highly resistant bacteria. Against eight clinical VIM-producing *K. pneumoniae* and *E. cloacae*, we demonstrated that aztreonam/avibactam and meropenem combined with amikacin or plazomicin exhibit bactericidal activity following 24 hours in a static time-kill assay. This activity was achieved using clinically achievable concentrations of these agents and thus can provide guidance in treating patients with VIM-producing CRE infections. More clinical data are necessary to validate these findings in patients.

## SUPPLEMENTAL MATERIAL

Supplemental material spectrum.03876-23-s0001.docx Tables S1 to S3; Fig. S1 and S2.

ASM does not own the copyrights to Supplemental Material that may be linked to, or accessed through, an article. The authors have granted ASM a non-exclusive, world-wide license to publish the Supplemental Material files. Please contact the corresponding author directly for reuse.
