# Supplementary material for: Comparing the activity of broad-spectrum beta-lactams in combination with aminoglycosides against VIM-producing Enterobacteriaceae
Source: Microbiol Spectr. 2024 Aug 20;12(10):e03876-23. doi: 10.1128/spectrum.03876-23 (PMC11448446; doi:10.1128/spectrum.03876-23)
Supplement: Supplemental material — Tables S1 to S3; Fig. S1 and S2. [file spectrum.03876-23-s0001.docx]

**Supplemental Materials**

**Tables**

| **Supplemental Table 1:** Change in Bacterial Concentration for All Sampled Time Points | | | |
| --- | --- | --- | --- |
| **Drug Conditions** | **T4** | **T8** | **T24** |
| **AMK** | -0.97 ± 1.6 | 0.43 ± 1.95 | 3.98 ± 0.24 |
| **ATM-AVI** | -2.91 ± 0.53 | -3.65 ± 0.23 | -3.86 ± 0.09 |
| **ATM** | 0.77 ± 2.92 | 2.12 ± 2.6 | 3.04 ± 2.75 |
| **FEP** | -1.8 ± 0.81 | 0.96 ± 2.66 | 3.75 ± 0.86 |
| **FEP-AMK** | -2.17 ± 0.24 | -3.11 ± 0.6 | -0.82 ± 3.37 |
| **FEP-PLZ** | -2.66 ± 1.61 | -2.77 ± 1.49 | -3.16 ± 1.05 |
| **GC** | 3.99 ± 0.3 | 4.14 ± 0.1 | 4.14 ± 0.1 |
| **MEM** | -3.44 ± 0.41 | -3.42 ± 0.86 | -2.3 ± 2.7 |
| **MEM-AMK** | -3.03 ± 0.44 | -3.84 ± 0.16 | -3.89 ± 0.1 |
| **MEM-PLZ** | -3.11 ± 1.07 | -3.32 ± 0.92 | -3.64 ± 0.45 |
| **PLZ** | -2.67 ± 1.62 | -2.76 ± 1.49 | -3.11 ± 1.09 |
| **TZP** | 1.11 ± 2.05 | 4.09 ± 0.14 | 3.99 ± 0.2 |
| **TZP-AMK** | -1.8 ± 0.31 | -1.9 ± 1.48 | 1.53 ± 2.98 |
| **TZP-PLZ** | -2.63 ± 1.54 | -2.77 ± 1.42 | -3.21 ± 0.96 |
| Bacterial concentrations (Log_10_ CFU/mL) are reported as geometric mean ± standard deviation.  **AMK:** amikacin, **ATM-AVI:** aztreonam/avibactam, **ATM:** aztreonam, **FEP:** cefepime, **FEP-AMK:** cefepime/amikacin, **FEP-PLZ:** cefepime/plazomicin, **GC:** growth control, **MEM:** meropenem, **MEM-AMK:** meropenem/amikacin, **MEM-PLZ:** meropenem/plazomicin, **PLZ:** plazomicin, **TZP:** piperacillin/tazobactam, **TZP-AMK:** piperacillin/tazobactam/amikacin, **TZP-PLZ:** piperacillin/tazobactam/plazomicin. | | | |

| **Supplemental Table 2:** Comparison of Bacterial Concentration Changes between Amikacin- and Plazomicin-containing Exposures after 24 Hours | | | |
| --- | --- | --- | --- |
| **Group 1** | **Group 2** | **Mean Difference [95% CI]** | **P value** |
| **AMK** | **PLZ** | 7.09 [4.14, 10.03] | < 0.001 |
| **FEP-AMK** | **PLZ** | 2.28 [-0.66, 5.23] | 0.492 |
| **MEM-AMK** | **PLZ** | -0.78 [-3.73, 2.16] | 1 |
| **TZP-AMK** | **PLZ** | 4.54 [1.59, 7.49] | < 0.001 |
| **AMK** | **FEP-PLZ** | 7.14 [4.19, 10.09] | < 0.001 |
| **FEP-AMK** | **FEP-PLZ** | 2.34 [-0.61, 5.28] | 0.435 |
| **MEM-AMK** | **FEP-PLZ** | -0.73 [-3.68, 2.21] | 1 |
| **TZP-AMK** | **FEP-PLZ** | 4.59 [1.65, 7.54] | < 0.001 |
| **AMK** | **MEM-PLZ** | 7.63 [4.68, 10.57] | < 0.001 |
| **FEP-AMK** | **MEM-PLZ** | 2.82 [-0.13, 5.77] | 0.086 |
| **MEM-AMK** | **MEM-PLZ** | -0.25 [-3.19, 2.7] | 1 |
| **TZP-AMK** | **MEM-PLZ** | 5.08 [2.13, 8.02] | < 0.001 |
| **AMK** | **TZP-PLZ** | 7.26 [4.31, 10.21] | < 0.001 |
| **FEP-AMK** | **TZP-PLZ** | 2.45 [-0.49, 5.4] | 0.316 |
| **MEM-AMK** | **TZP-PLZ** | -0.61 [-3.56, 2.33] | 1 |
| **TZP-AMK** | **TZP-PLZ** | 4.71 [1.76, 7.66] | < 0.001 |
| Mean difference was calculated as Group 1 – Group 2. Groups compared aggregated bacterial concentration (Log_10_ CFU/mL) differences across each respective experiment.  **AMK:** amikacin, **FEP-AMK:** cefepime/amikacin, **FEP-PLZ:** cefepime/plazomicin, **MEM-AMK:** meropenem/amikacin, **MEM-PLZ:** meropenem/plazomicin, **PLZ:** plazomicin, **TZP-AMK:** piperacillin/tazobactam/amikacin, **TZP-PLZ:** piperacillin/tazobactam/plazomicin. | | | |

| **Supplemental Table 3:** Comparison of Bacterial Concentration Changes between Aztreonam/Avibactam and Aminoglycoside-containing Exposures after 24 Hours | | | |
| --- | --- | --- | --- |
| **Group 1** | **Group 2** | **Mean Difference [95% CI]** | **P value** |
| **ATM-AVI** | **AMK** | -7.85 [-10.79, -4.9] | < 0.001 |
| **ATM-AVI** | **FEP-AMK** | -3.04 [-5.99, -0.1] | 0.032 |
| **ATM-AVI** | **MEM-AMK** | 0.03 [-2.92, 2.97] | 1 |
| **ATM-AVI** | **TZP-AMK** | -5.3 [-8.24, -2.35] | < 0.001 |
| **ATM-AVI** | **PLZ** | -0.76 [-3.7, 2.19] | 1 |
| **ATM-AVI** | **FEP-PLZ** | -0.71 [-3.65, 2.24] | 1 |
| **ATM-AVI** | **MEM-PLZ** | -0.22 [-3.17, 2.73] | 1 |
| **ATM-AVI** | **TZP-PLZ** | -0.59 [-3.53, 2.36] | 1 |
| Mean difference was calculated as Group 1 – Group 2. Groups compared aggregated bacterial concentration (Log_10_ CFU/mL) differences across each respective experiment.  **AMK:** amikacin, **ATM-AVI:** aztreonam/avibactam, **FEP-AMK:** cefepime/amikacin, **FEP-PLZ:** cefepime/plazomicin, **MEM-AMK:** meropenem/amikacin, **MEM-PLZ:** meropenem/plazomicin, **PLZ:** plazomicin, **TZP-AMK:** piperacillin/tazobactam/amikacin, **TZP-PLZ:** piperacillin/tazobactam/plazomicin. | | | |

**Figures**

| 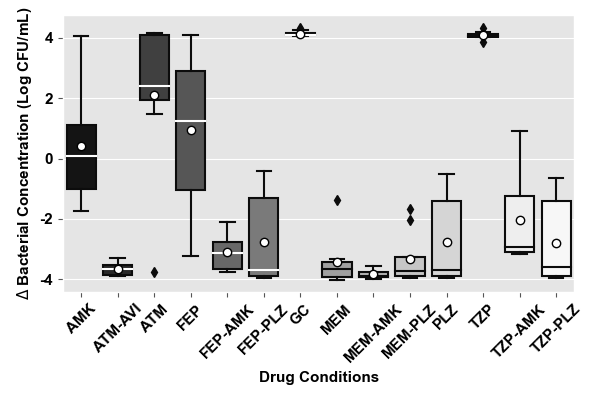 |
| --- |
| **Supplemental Figure 1:** Above are the boxplots illustrating the distributions of log bacterial reduction following 8 hours of exposure. Outliers are denoted as filled diamonds above/below the upper/lower fence, and geometric means are denoted as white circles.  **AMK:** amikacin, **ATM-AVI:** aztreonam/avibactam, **ATM:** aztreonam, **FEP:** cefepime, **FEP-AMK:** cefepime/amikacin, **FEP-PLZ:** cefepime/plazomicin, **GC:** growth control, **MEM:** meropenem, **MEM-AMK:** meropenem/amikacin, **MEM-PLZ:** meropenem/plazomicin, **PLZ:** plazomicin, **TZP:** piperacillin/tazobactam, **TZP-AMK:** piperacillin/tazobactam/amikacin, **TZP-PLZ:** piperacillin/tazobactam/plazomicin. |

| 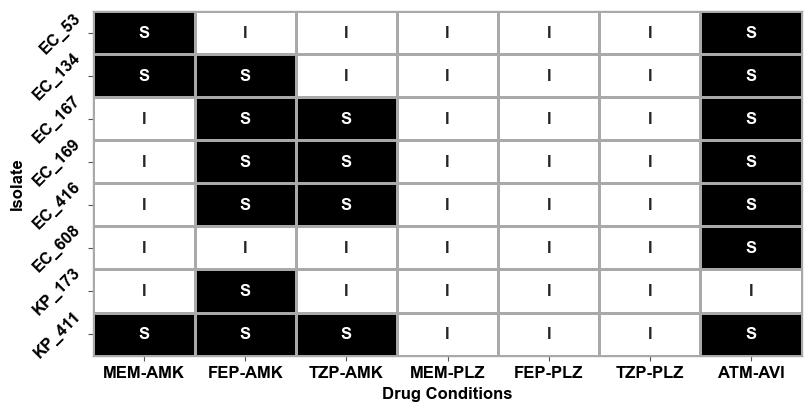 |
| --- |
| **Supplemental Figure 2:** Illustrated is a heatmap showing the categorical synergy analysis for each drug combination against all isolates tested. The average bacterial reductions measured across all duplicate trials for each isolate were utilized for this assessment.  **S:** synergistic, **I:** indifferent, **ATM-AVI:** aztreonam/avibactam, **FEP-AMK:** cefepime/amikacin, **FEP-PLZ:** cefepime/plazomicin, **MEM-AMK:** meropenem/amikacin, **MEM-PLZ:** meropenem/plazomicin, **TZP-AMK:** piperacillin/tazobactam/amikacin, **TZP-PLZ:** piperacillin/tazobactam/plazomicin. |
